# Unraveling Childhood Obesity: A Grounded Theory Approach to Psychological, Social, Parental, and Biological Factors

**DOI:** 10.3390/children11091048

**Published:** 2024-08-28

**Authors:** Georgia Karakitsiou, Spyridon Plakias, Foteini Christidi, Anna Tsiakiri

**Affiliations:** 1 Department of Psychiatry, Medical School, Democritus University of Thrace, 68100 Alexandroupolis, Greece; gkarakitsiou@yahoo.gr; 2 Department of Physical Education and Sport Science, University of Thessaly, 38221 Trikala, Greece; spyros_plakias@yahoo.gr; 3 Department of Neurology, Medical School, Democritus University of Thrace, 68100 Alexandroupolis, Greece; atsiakir@med.duth.gr

**Keywords:** childhood obesity, family factors, societal factors, individual factors, grounded theory, qualitative research

## Abstract

Childhood obesity is a major medical and public health issue of global interest, which is influenced by a diverse array of factors and carries significant medical and psychosocial implications. Despite the extensive studies that have been conducted to explore the specific issue, the impact of several factors that influence, generate, worsen, and make chronic the phenomenon needs further exploration. This study aimed to construct a grounded theory that includes and connects the psychological, social, parental, and biological factors affecting childhood obesity. Key psychological factors include mental health issues such as depression and emotional eating, while social factors encompass socioeconomic status and cultural influences. Parental factors involve parenting styles and feeding practices, and biological factors relate to genetic predispositions and prenatal conditions. These factors interact in complex ways, highlighting the multifactorial nature of childhood obesity. The study employed a qualitative grounded theory approach, using research articles to achieve a thorough understanding. Qualitative analysis of the articles was conducted using Atlas.ti 24.0 software. Twenty-five research articles were required to reach theoretical saturation. The analysis resulted in 336 codes that were grouped into seven broad categories and twenty-four different subcategories. Through the construction of the theoretical framework, it was recognized that obesity in minors is a complex and multifactorial issue and that the network of causes and influencing factors covers a broad spectrum ranging from the individual to the family, and subsequently to society at large, which interact with each other.

## 1. Introduction

Childhood obesity is a major medical and public health issue of global interest, marked by an abnormal or excessive buildup of body fat that poses health risks and affects various systems. The most commonly used tool for the early diagnosis of obesity is the body mass index (BMI). However, BMI is influenced by growth dynamics and therefore cannot be used in the same way for children as it is for adults, as the BMI varies significantly with age [1]. Due to the aforementioned difficulties, experts often resort to other internationally recognized systems in order to categorize children based on their weight, such as the French system, known as the International Obesity Task Force (IOTF). In other cases, they may resort to the references from the World Health Organization (WHO) [2]. Both of these systems use specific growth curves based on data from different populations to determine the thresholds for weight categories, although they use different methods for calculating childhood obesity. According to the above, it seems quite logical that the absence of a universally agreed-upon definition of childhood obesity makes it challenging to determine which groups of children require treatment and which do not [3]. However, beyond the aforementioned challenges and complexities related to the identification of childhood obesity, what is widely recognized across scientific communities is that it constitutes a very serious phenomenon approaching epidemic proportions, demanding immediate attention [4].

Childhood obesity is influenced by a diverse array of factors as underscored in the scholarly literature [3,5]. These factors cover a broad spectrum and can be grouped into broader categories. The key influencing categories are: social factors such as the family’s socioeconomic status and economic parameters; psychological factors such as the prevalence of mental health issues; parental factors such as family meal habits; and biological factors such as prenatal conditions and the genetic specifications of the parents [6,7,8,9,10,11]. Addressing these multifaceted influences through targeted interventions at the individual, family, community, and policy levels is essential for tackling childhood obesity and fostering healthier societies [12,13]. This is considered almost imperative, as obesity represents a significant component of the worldwide challenge of chronic illness and disability, carrying substantial social and psychological consequences that impact individuals of all ages and socioeconomic backgrounds [5].

More specifically, childhood obesity carries significant medical and psychosocial implications. Medically, it is associated with metabolic conditions. Furthermore, obesity in children can significantly affect the functioning of the body’s vital organs, contribute to respiratory issues, polycystic ovarian syndrome, liver dysfunction, renal impairment, dental caries, and cardiovascular complications [4,14,15,16]. Psychosocially, children with obesity experience a wide range of significant consequences that affect their quality of life [17,18]. In particular, children with obesity often experience depression, social stigma, and diminished health-related quality of life. Early intervention is critical to prevent the development of obesity-related disorders in adulthood, underscoring the importance of addressing weight-related comorbidities and promoting healthy lifestyle habits from an early age [8,19]. Due to its severity, diverse causes, and the profound long-term consequences that may persist across generations, extensive research has been conducted on childhood obesity. These studies have investigated both its origins and its outcomes comprehensively, employing both quantitative and qualitative research methods to thoroughly examine this phenomenon [20,21,22,23,24,25,26,27,28,29,30,31,32,33,34,35,36].

Specifically, regarding qualitative research, various techniques have been applied to explore the phenomenon of childhood obesity. One of these techniques includes the use of grounded theory methodology. Grounded theory, as a qualitative research methodology, shows great potential for solving the complexities inherent in multifactorial issues. The aim of grounded theory is to construct a theoretical framework or a cohesive explanatory mechanism that explains the phenomena being studied. The application of grounded theory methodology includes analyzing and interpreting data that are mostly qualitative like observations, interviews, texts, and documents. Moreover, through the application of this methodology, fundamental factors and processes that impact the phenomenon are identified, and the developed theories are based exclusively on the data without being influenced by prior theoretical knowledge [37,38]. While grounded theory has been utilized in researching childhood obesity, no comprehensive study has identified all potential variables and factors influencing this phenomenon. Existing research using the grounded theory method has predominantly focused on perceptions held by parents, children, or other groups regarding this issue. Alternatively, studies have explored specific demographic groups [39], geographic locations [40,41] or particular factors such as parental roles affecting childhood obesity [42,43,44,45] or parenting perspectives regarding the issue [46].

Recognizing this gap, an attempt was made to construct a theoretical framework that comprehensively investigated all potential influences and factors affecting childhood obesity as well as the interconnection between these factors. Additionally, efforts were made to categorize these factors into broader structures and determine the link between them through the development of a definitive and holistic model based exclusively on the foundational principles of grounded theory. The purpose of the study was to construct a grounded theory that includes and connects the psychological, social, parental, and biological factors that affect childhood obesity.

## 2. Materials and Methods

### 2.1. Methodology

In our research, a grounded theory approach was selected for the qualitative analysis due to its highly structured nature and well-established reputation [47,48]. The execution of this study adheres to the recommendations of Strauss and Corbin [49] and Glaser and Strauss [38]. In line with grounded theory principles, the hypotheses were not predetermined, but emerged and were tested throughout the analysis process [50]. Grounded theory is an inductive method, systematically collecting and analyzing data to develop theories based on them. The researchers of this particular study emphasized generating theory from the ground up, rooted in the data itself rather than starting with pre-existing theories or hypotheses, through rigorous analysis and coding techniques. The inductive nature of grounded theory allowed the researchers to derive insights and develop theories that are firmly grounded in the data, contributing to a deeper understanding of the social processes identified behind the phenomenon of childhood obesity. To conduct the present research, the data used to derive the theory were sourced from published research articles, which were found in the Scopus and Web of Science research databases [51,52,53].

Data were collected until category saturation was reached, meaning that the categories were well-defined, relationships between categories were clearly outlined, and additional data did not contribute new information. The dynamic phases of the grounded theory approach included (1) gathering rich and descriptive data, (2) using empirically-based coding and analysis procedures to identify themes, continually reflecting on the researcher’s perspective and its relationship to the data, and (3) generating theory that described these experiences and processes. The phases of qualitative data collection and analysis occurred simultaneously. The researchers worked separately to develop their codes. Subsequently, during a dedicated meeting, they collectively agreed upon the final coding. This process was followed to ensure the reduction in biases and prejudices. Furthermore, the researchers attempted to mitigate the influence of their personal beliefs and perceptions on the research topic to the extent that it was feasible. The analysis followed a process known as “constant comparison”, which means initially coding the data line-by-line and subsequently conducting more abstract coding to identify themes within the transcripts. This method enabled the researchers to discern similarities and differences within the data [38,50,54,55].

The meticulous search for the final bibliography used as data and the systematic application of grounded theory methods ensured that the final grounded theory that emerged achieved empirical grounding. The theory and its concepts were shown to closely align with the data, demonstrating credibility and trustworthiness [49]. Finally, it should be noted that ChatGPT was utilized for English language editing. Furthermore, the diagrams were created using draw.io, a free and open-source web-based application that enables users to create various types of diagrams [56].

### 2.2. Inclusion-Exclusion Criteria

Only articles written in English and found in the Scopus or Web of Science research databases to which the authors could have full text access were included. The Scopus and Web of Science databases were chosen because they provide extensive and international coverage of the literature. Additionally, they not only ensure a high quality of content and broad coverage of various scientific fields, but also continuous updating of the literature. Articles that were not accessible to the researchers or were not exclusively related to the issue of childhood obesity but extended to adult populations as well were not included. It is important to mention that no restrictions were applied during the literature search regarding the publication date of the articles. Finally, no specific restriction was applied regarding whether the included studies were quantitative or qualitative.

### 2.3. Search

The search for the files to be used as data was conducted on 30 May 2024 using the search engines Scopus and Web of Science. The BOOLEAN expression “(psychological OR psychology OR mental) AND (social OR psychosocial OR environmental OR cultural OR behavioral) AND (parental OR parents) AND style AND (biological OR genetic) AND (child OR minor OR childhood OR adolescent) AND obesity” was used in the titles, abstracts, and keywords of the articles. The initial search yielded thirty-four articles on Scopus and five on Web of Science. After the removal of duplicates, thirty-five articles remained. Two articles were removed because they were written in another language, three because they were not accessible, and two because they did not focus on childhood obesity and their purpose was different from the subject of our research. From the remaining articles that met the inclusion/exclusion criteria, twenty-five were ultimately necessary until theoretical saturation occurred, and these were used as data to build the theory. The search for articles was carried out by the primary and senior authors. Any disagreements were resolved during a meeting involving all authors.

### 2.4. Building the Grounded Theory

A grounded theory model for the factors that influence, generate, exacerbate, and make chronic the phenomenon of childhood obesity was developed from the analysis of the data using the recommendations made by Corbin and Strauss [50] and Glaser and Strauss [38]. The 25 articles were imported into the ATLAS.ti version 24.0 (Atlas.ti GmbH, Berlin, Germany) software for which the first author holds a nominal license, where an independent comprehensive study of the articles and content analysis were conducted by the first two authors.

Regarding the coding process, the provisional initial codes were meticulously compared both with each other and the data itself. Through this iterative process of coding and constant comparison, these codes were further developed, elaborated upon, and grouped together based on the similarities and differences, and conditional/consequential matrices were constructed. This refinement led to the creation of fewer but more focused and comprehensive codes. Open coding was used to identify and categorize thought units from the data independently by the first two authors, followed by collaborative analysis with all of the researchers to establish consensus on the themes and concepts, which were discussed and agreed in a joint meeting of all authors [38,50,57].

Ultimately, the constructed focused codes aligned closely with the data, reflecting a thorough and systematic approach to data analysis (focused coding). In another meeting with all authors, the final extraction of key concepts, categories, subcategories, and their connections as well as the identification of relationships between categories was performed (axial coding). During this meeting, the final conclusions for theory construction were drawn, and the theoretical model was refined based solely on the data (selective coding). This iterative process led to the development of a comprehensive theoretical framework through multiple revisions and critical reflections, ensuring a grounded understanding of the study’s findings. Furthermore, this iterative process helped in the construction of major categories, which are called “core concepts” of the study [58,59].

## 3. Results

The authors, year of publication, and title of the 25 articles that were used as data for the extraction of the theory are included in Table 1. The articles that follow are alphabetically sorted.

The research identified seven different categories, twenty-four different subcategories, and three hundred and thirty-six unique codes that constitute risk factors for the development of childhood obesity and factors that influence, generate, exacerbate, and make chronic the phenomenon of childhood obesity.

**Figure 1:**
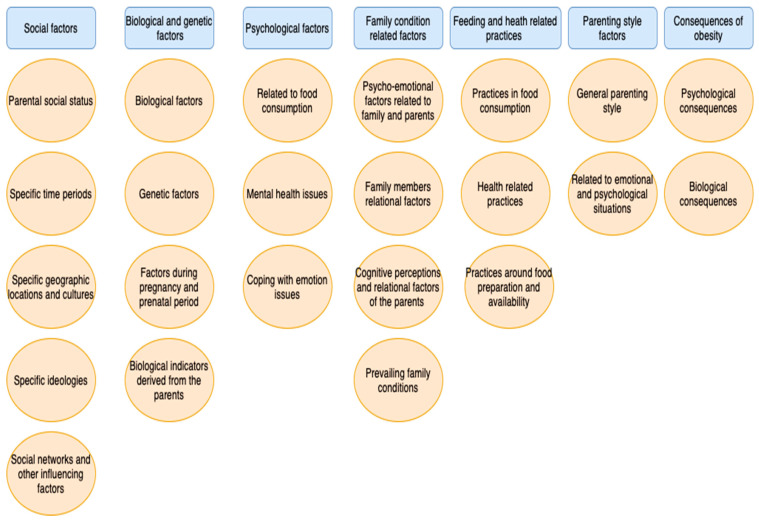
Diagram presenting the main categories with their subcategories.

In the following diagram (Figure 1), the main categories with their subcategories are presented. The main categories are depicted in blue and rectangular frames, while the subcategories are depicted in pink and circular frames. In Appendix A, all codes are additionally presented, classified into the corresponding categories and subcategories.

## 4. Discussion

### 4.1. Social Factors

The first category that emerged was “social factors (1)”. The first subcategory was “parental social status”. It was found that social inequalities and low socioeconomic status of the parents were associated with a higher likelihood of obesity. Regarding the subcategory “social factors related to specific time periods”, it was concluded that prevailing conditions during specific periods, such as the time when COVID-19 was dominant, can affect dietary habits, and consequently the individuals’ weight. As for the subcategories “social status related to specific geographic locations and cultures” and “social status related to specific ideologies”, it became evident that the country where someone was born or the cultural group to which they belonged could significantly influence dietary habits that favor obesity. Finally, the subcategory “social networks and other influencing factors” highlighted that the influence of social networks, relationships with peers, and an individual’s social life can be strong predictors for the onset of obesity. In summary, it can be said that not only may the social status of a family, but also broader geopolitical and temporal factors, be related to and significantly influence the phenomenon of childhood obesity, elevating it to a broader social issue rather than just an individual or familial problem.

As demonstrated by the relevant literature, children whose parents had a lower social status were more likely to be overweight/obese compared to children not at risk [68]. Moreover, it has been proven that the pre-adolescent BMI is associated with the social environment [63], and that social vulnerabilities exacerbate or buffer the effect on different lifestyles and stress connected to childhood obesity [85]. The impact of psychosocial factors and other environmental influences that are associated with prevailing social and seasonal conditions determining whether a child becomes obese has also been highlighted by other studies [62]. Finally, through the literature review, a significant influence of sociocultural conditions on the prevalence and persistence of childhood obesity is highlighted [60,64,76,81].

### 4.2. Biological-Genetic Factors

The second category was “biological-genetic factors (2)”. This categorization revealed a multitude of biological and genetic markers associated with childhood obesity, some of which may originate from the perinatal period or stem from the direct effects of parental genes, extending the scope of the phenomenon to previous generations. The first subcategory, “biological factors”, includes individual biological markers such as mechanisms of metabolic programming, hormonal signaling, and biological transgenerational effects like heredity. The subcategory “genetic factors” refers to individual, unchangeable factors linked to obesity such as age, gender, and genomic characteristics. The subcategory “factors during pregnancy and the prenatal period” highlights several prenatal factors that may be related to childhood obesity such as maternal weight gain during pregnancy, maternal obesity in the first trimester, excess maternal weight prior to conception, and diet and physical activity habits during pregnancy. Finally, the subcategory “biological indicators derived from the parents” describes parental biological characteristics that may contribute to the development of childhood obesity such as abnormal body mass in at least one parent, parental slimness in childhood, and the parents’ diet, taste, and nutritional preferences.

It is widely recognized in the scientific community how significant the influence of genes and genetic and biological factors is in creating a strong predisposition for childhood obesity, which usually manifests in combination with the impact of environmental factors. This has been evidenced by numerous studies and research. Holmen et al. [67] studied genetic and environmental interactions through generations while Murrin et al. [75] conducted an analysis of genotypic and phenotypic data over three generations in order to understand the nature of the maternal–offspring relationship. There have been a plethora of studies examining the genetic and biological factors, which in combination with other factors create the intergenerational cycle of obesity [61,66]. However, there are also studies that have focused exclusively on the influence of genetic factors such as one conducted by Faith et al. [65], which highlighted the significant impact of gender on the phenomenon of childhood obesity by using boys and girls as the sample for the research, although the effects identified were only seen in boys.

### 4.3. Psychological Factors

The category “psychological factors (4)” is divided into three subcategories: “related to food consumption”, “mental health issues”, and “coping with emotional issues”. This category highlights psychological mechanisms that emotionally burden individuals, making them vulnerable to the development of childhood obesity. Through the literature review, a multitude of factors emerged ranging from psychiatric issues to behaviors involving emotional stress related to food consumption. The subcategory “related to food consumption” encompasses behaviors tied to the emotional aspects of eating that contribute to childhood obesity such as binge eating, emotional feeding by parents, inability to monitor food intake, emotional eating, or eating in the absence of hunger. The subcategories “mental health issues” and “coping with emotional issues” address emotional or psychological problems that are often linked to the development of obesity including anxiety, depression, increased levels of negative affect, lower emotional awareness, or difficulty in managing negative emotions.

The impact of the psychological factors on the onset, exacerbation, and maintenance of childhood obesity was studied by Poulain et al. [78], who investigated, above all, the psychological assessments for children and parents that were related to childhood obesity in the LIFE Child study. This study is a large population-based longitudinal childhood cohort study conducted in the city of Leipzig, Germany. Moreover, Grube et al. [86] focused on the psychological and psychiatric factors of both the parents and children, which may constitute risk factors for the development of childhood obesity. In most of the studies used in this research, however, psychological mechanisms related to food consumption by minors and childhood obesity also emerged. It appears that these psychological mechanisms lead to behaviors that maintain or exacerbate the phenomenon.

### 4.4. “Family Condition-Related Factors”, “Parenting Style Factors”, and “Feeding and Health Related Practices”

The categories “family condition-related factors (3)”, “parenting style factors (5)”, and “feeding and health related practices (6)” will be discussed and presented together here, as all three of the above categories move away from the broader social, genetic, or individual context and focus their attention on the family level, treating it as a whole in relation to the persistence and unsuccessful management of childhood obesity.

More specifically, the category “family condition-related factors (3)” consists of the following subcategories: “psycho-emotional factors related to family and parents”, which refers to conditions such as maternal stress, the inability of parents to regulate negative emotions like sadness and stress, and child maltreatment, all of which are documented as being related to childhood obesity. The subcategories “family members’ relational factors” and “prevailing family conditions” indicate that difficulties in relationships among family members, poor family functioning, a negative emotional climate during meals, poor communication, parental separation or divorce, and other factors related to the prevailing family environment can be strong predictors of childhood obesity. Finally, the subcategory “cognitive perceptions and relational factors of the parents” refers to how low parental concern about their child’s thinness, difficulty in recognizing weight problems, parental perceptions of diet, and other related factors may affect their child’s weight.

The category “parenting style factors (5)” consists of “general parenting style” and “parenting style related to emotional and psychological situations”. These subcategories suggest that factors such as a strict, permissive, authoritarian, neglectful, or uninvolved parenting style, insecure attachment relationships, lack of acceptance from parents, and similar situations may strongly influence children’s weight.

Finally, the category “feeding and health-related practices (6)” is divided into “practices in food consumption”, “health-related practices”, and “practices around food preparation and availability”. All of these subcategories indicate factors such as poor eating habits like not drinking enough water or not chewing food adequately, lack of assistance during mealtimes, sleep deprivation, exposure to certain foods after a period of restriction, pressuring children to eat, not promoting physical activity, not controlling screen time, not educating children about nutrition, and not offering diverse food choices. These and other similar factors are closely related to the development of childhood obesity.

Through this specific categorization, it is clear that the family constitutes a key pillar through which programs and interventions to address childhood obesity can be designed. The family’s influence on the creation, progression, and maintenance or resolution of the phenomenon is of paramount importance. This has been confirmed and validated by numerous research studies that have thoroughly examined the family’s influence on the phenomenon of childhood obesity and have consistently identified strong effects and correlations [62,69,70,71,72,73,74,77,79,83]. In almost all of the above studies, a plethora of factors related to childhood obesity and connected with the parental role have been identified, and these factors are diverse. These may relate to the exercise of parental roles themselves, cognitive perceptions, or multiple other factors related to parents. The importance of intrafamily relationships also emerged as well as the warmth and emotional responsiveness of the parent toward their child, serving as a core and central axis in managing the phenomenon of childhood obesity.

### 4.5. Consequences of Obesity

The last category is “consequences of obesity (7)”, which includes “social consequences” such as weight-related stigma, body image concerns, being avoided, ignored, or the subject of negative rumors; “psychological consequences” like emotional difficulties, mental disorders, higher rates of sadness, loneliness, and anxiety as well as decreased self-esteem; and “biological consequences” including increased mortality, cardiovascular and metabolic disorders, various types of cancer, and more. This category underscores the severity of childhood obesity, highlighting its multifaceted consequences and the broad spectrum of its impacts. This understanding helps to mobilize efforts to address the issue and improve the quality of life of those affected by childhood obesity.

Paul et al. [77] suggested the importance of early life experiences on long-term health trajectories. Meanwhile, Van De Beek et al. [82] investigated the effects of childhood obesity on cardiovascular, physical and mental health, diet and physical activity measures, child growth and development measures, biological samples, and genetic and epigenetic information. Similarly, Vedanthan et al. [83] focused their attention on the consequences of obesity on cardiovascular diseases.

It is important to emphasize, however, that in almost every study used for the present research, there were references related to the consequences of childhood obesity, even in cases where this was not the purpose of the study. This fact led to the creation of a category related to the consequences of childhood obesity, as these consequences elevated it to an extremely important factor related to the maintenance and chronicity of the phenomenon. It was found that the consequences of childhood obesity cover an exceptionally wide range including social marginalization, emotional burden, and biological impact.

### 4.6. Grounded Theory

**Figure 2:**
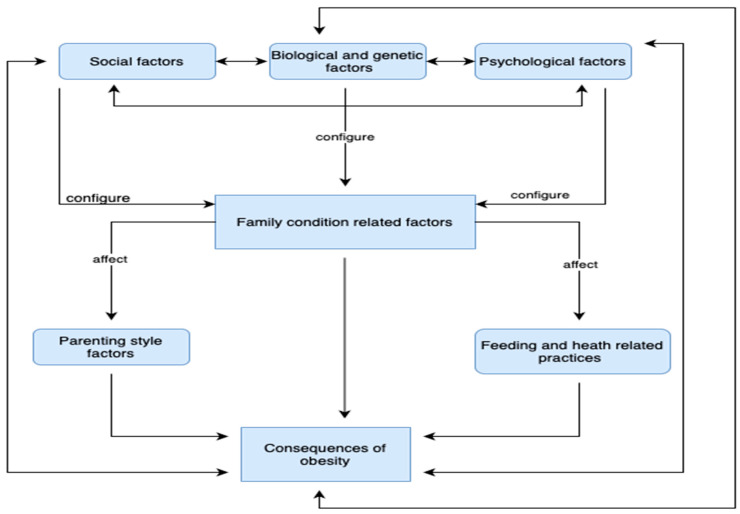
Diagram of the grounded theory on the impact of the psychological, social, parental, and biological factors that affect childhood obesity.

In the present research, an extensive examination of the psychological, social, parental, and biological factors that interact with each other regarding the complex phenomenon of childhood obesity was conducted, revealing a multitude of causations and effects. The research identified seven different categories, twenty-four different subcategories and three hundred and thirty-six unique codes that constitute risk factors for the development of childhood obesity. The interconnection of these seven concepts can form the basis for a grounded theory explaining how psychological, social, and genetic-biological factors affect not only the maintenance of childhood obesity, but also the perpetuation and deterioration of the phenomenon over the years. The basic concept is that social factors (1), biological-genetic factors (2), and psychological factors (4) constitute a strong starting point and are the main factors that influence family condition-related factors (3). All of the above influence parenting style factors (5) and feeding and health related practices (6). Moreover, the above six concepts interconnect dynamically with the category of the consequences of the obesity (7), which leads to the perpetuation of the phenomenon of childhood obesity. The core structure of the grounded theory is depicted in Figure 2.

A deeper analysis of the identified subcategories revealed that social factors (1) covered a broad spectrum including social inequalities, low socioeconomic status of parents, prevailing conditions during specific periods, and other influential factors such as the country of birth, cultural group, peer relationships, social life, and broader geopolitical and seasonal factors. These elements significantly impact childhood obesity, elevating it to a broader social issue rather than merely an individual or familial problem.

The aforementioned spatiotemporal social influences interact directly with biological-genetic factors (2). These factors relate to an individual’s biological markers, unchangeable aspects such as age, gender, and genomic characteristics, prenatal factors, and biological traits of parents that may contribute to childhood obesity. Categories (1) and (2) also interact with psychological factors (4), which involve psychological mechanisms that emotionally burden individuals, increasing their vulnerability to childhood obesity. These categories form a strong foundation and are the primary influencers of childhood obesity, spanning societal, familial, and individual levels.

The interaction among these three categories helps shape family condition-related factors (3). These include the inability of parents to regulate their negative emotions, relationship difficulties among family members, poor family functioning, negative emotional climates during meals, poor communication, parental separation or divorce, and other factors related to prevailing family conditions. According to the revelation of the above mechanism, it is highlighted that the society in which someone is born and raised, combined with their individual and familial genetic and psychological characteristics, influences the conditions within a family context, affecting the occurrence or non-occurrence of childhood obesity.

Delving further, it seems that family condition factors significantly impact parenting style factors (5), which directly influence the likelihood of developing childhood obesity. Factors such as strict, permissive, neglectful, or uninvolved parenting style, insecure attachment relationships, and lack of parental acceptance can strongly affect the children’s weight. Additionally, the interaction among categories (1), (2), (3), and (4) also affects feeding and health-related practices (6). This category encompasses family eating or dietary habits and the stance parents take, both nutritionally and behaviorally, toward their children to either promote or prevent childhood obesity. It should also be noted that categories (5) and (6) are interrelated and strongly connected to each other.

Finally, all six concepts dynamically interconnect with the category of the consequences of obesity (7). This category includes social, psychological, and biological factors. The consequences of obesity tend to persist over time, primarily affecting the individual, but also extend their influence to the family and society later on. Therefore, it appears that the seventh category primarily influences categories (1), (2), and (4), and secondarily impacts categories (3), (5), and (6), forming a complex spiral of dynamics and interactions among the factors, categories, and subcategories.

Through the research, a strong interconnection among all categories emerged. For example, Russel and Russel [80] suggested a biopsychosocial model that included three primary influences on developmental outcomes, which were constitutional, genetic, or biological characteristics, social, psychological, and behavioral environment, with a focus on parenting, parent–child relationships, and the family environment, and finally, the environmental, societal, and cultural factors. According to their theory, whether a biologically-based risk results in a developmental outcome depends on the environmental conditions and psychosocial factors, particularly those associated with family and parenting. Similarly, Mazzeo et al. [73] revealed that although genetic factors appear to contribute to obesity risk, they have also been hypothesized to be associated with an obesogenic or “toxic” environment, which deeply affects physical and psychological health.

Moreover, Oparaocha [76] revealed the interaction effects among environmental factors, genetic predisposition, and individual behavior on excess weight gain. Furthermore, Carnell et al. [61] highlighted this connection, supporting that individuals come into the world genetically and epigenetically loaded with powerful biologically influenced predispositions toward food, which are captured in our biology, and particularly in our brains. Environmental factors can certainly affect these dispositions, but often these influences are overwhelmed by the expression of innate tendencies, and environmental factors correlate with appetitive behavior. Findings from other research are consistent with the findings above-mentioned [63,78].

**Figure 3:**
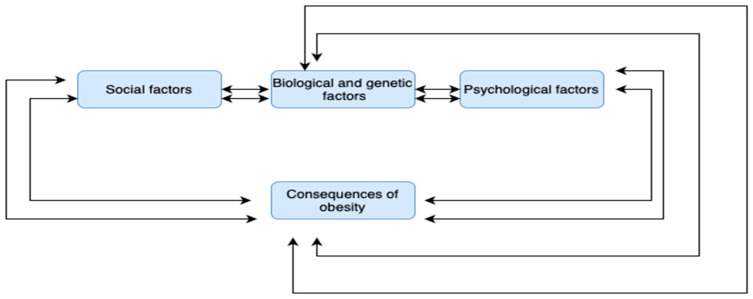
Diagram of the first key sub-relationship that emerged.

The seven concepts discussed were not only interconnected, but it was also found that some of the categories were linked to each other with stronger correlations compared to others. For example, social factors (1) interact with biological and genetic factors (2) and with psychological factors (4), leading to the significant consequences of childhood obesity (7), which in turn burden individuals in terms of biological, social, and psychological factors, creating a vicious cycle of obesity that perpetuates itself, passing from one generation to the next. This sequence is supported by Haire-Joshu and Tabak [66], who emphasized that the intergenerational transmission of social disadvantage and obesity may be partially explained by epigenetic changes in gene expression that are passed across generations. Additionally, this relationship is much better explained through the mechanism of “gene–environment interaction”, which refers to a situation in which the response or adaptation to an environmental agent, behavior, or a change in behavior interacts with the genotype of the individual [76]. According to the above, the hypothesis is confirmed that the severe consequences of childhood obesity, which significantly burden individuals at the psychological, social, and biological level, make them even more prone not only to maintaining obesity, but also to perpetuating it from generation to generation. The first key sub-relationship that emerged is shown in Figure 3. The relationships between the categories are depicted with two arrows to indicate that the correlation is stronger between the aforementioned categories.

**Figure 4:**
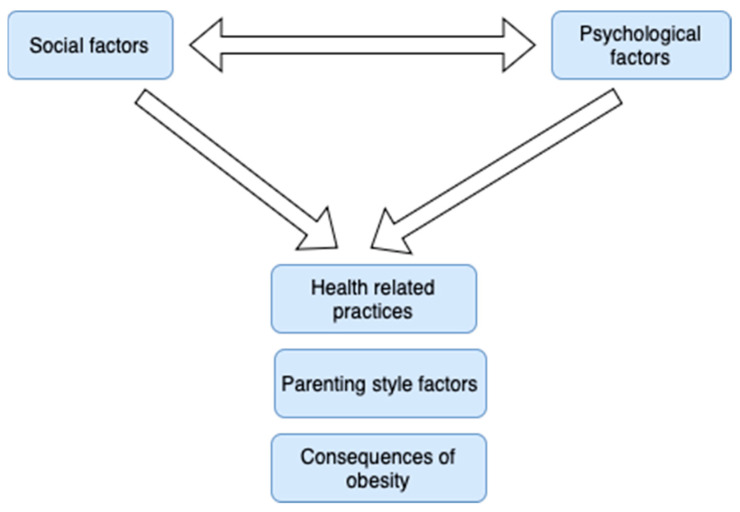
Diagram of the second key sub-relationship that emerged.

Additionally, further interconnections among some subcategories can be highlighted. For example, social factors (1) influence psychological factors (4), and the interaction between the above two leads to the formation of parenting style factors (5), health related practices (6), and the consequences of obesity (7). Similarly, Iguacel et al. [85] showed that an interaction between socioeconomic adversity, family disharmony, and childhood stress led to unhealthy behaviors and finally overweight and obesity. Moreover, it has been stated that social vulnerabilities tend to intensify the exposure to obesity-promoting influences [68]. Haire-Joshu and Tabak [66] highlighted through their research that sociocultural dimensions may influence early life obesity through a number of pathways that impact how a parent feeds a child. They argued that a complex combination of parental attitudes and beliefs, family dynamics, and socioeconomic factors influence complementary feeding practices and partially explain the early obesity risk. Similar findings that reinforce the interconnection between the above categories have emerged [84]. The second key sub-relationship that emerged is shown in Figure 4.

**Figure 5:**
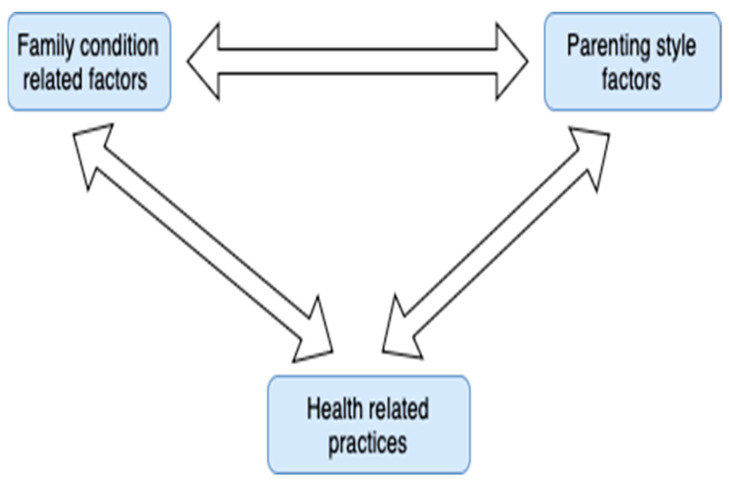
Diagram of the third key sub-relationship that emerged.

In general, it has been proven that family dynamics are essential to address the foundation for behavior change and the promotion of healthy behaviors [66]. According to the above, a very strong interaction emerged between family condition-related factors (3), parenting style factors (5), and health related practices (6). Rusell and Russel [80] revealed that parental cognitions are important in how parents respond to and interact with children regarding food and eating. The above finding has also been highlighted by other studies [71,72]. Similarly, Ji and An [69] stated that the family provides an environmental and emotional context for children’s development and considerably influences the children’s health including childhood obesity risk factors across their life course. The above research findings highlight strong connections among the subcategories family condition-related factors (3), parenting style factors (5), and health related practices (6) in the same manner as those supported by the present grounded theory. The third key sub-relationship that emerged is shown in Figure 5.

**Figure 6:**
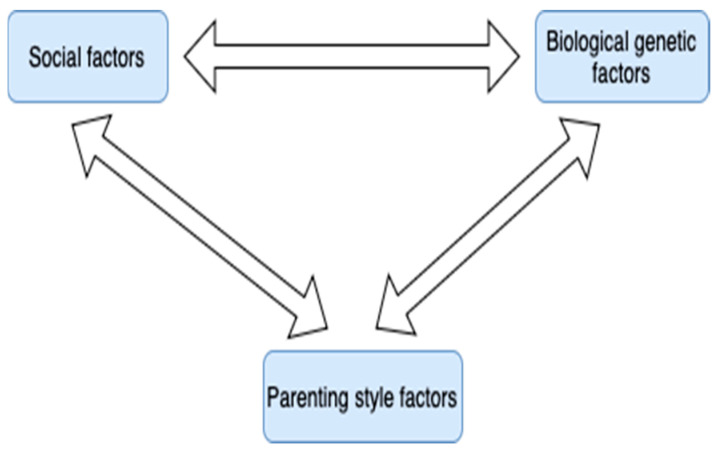
Diagram of the fourth key sub-relationship that emerged.

Finally, through research, direct connections were identified between the categories social factors (1), biological-genetic factors (2), and parenting style factors (5). For example, Ji and An [70] hypothesized that parenting styles served as a buffer and moderated the genetic and environmental influences on childhood obesity. In another instance, direct connections were identified between the categories social factors (1), biological-genetic factors (2), and feeding and health-related practices (6), as it is suggested that biological factors such as genetic predispositions and temperament as well as psychosocial factors influence feeding styles and feeding practices [80]. The fourth key sub-relationship that emerged is shown in Figure 6.

## 5. Conclusions

This study synthesized an array of studies to construct a grounded theory elucidating the factors that influence, generate, exacerbate, and make chronic the complex and multifactorial phenomenon of childhood obesity. Central to this framework is the recognition that obesity in minors is an exceedingly complex and multifactorial issue. Only by comprehending the mechanisms behind this specific phenomenon and by elucidating the spiral of interactions among them can appropriate measures be devised and implemented successfully to address it. According to the above, a network of seven factors related to childhood obesity was constructed: social factors (1), biological-genetic factors (2), family condition-related factors (3), psychological factors (4), parenting style factors (5), feeding and health related practices (6), and consequences of obesity (7). This network covers a broad spectrum ranging from the individual to the family, and subsequently, to society at large.

However, limitations exist within the study’s scope. Firstly, the grounded theory approach, while insightful, precludes the direct causality inference that quantitative studies might afford [54,87]. In particular, the final outcomes rely on the researcher’s interpretation of the data, and the coding process involves subjective decisions made by researchers. Furthermore, it is important to note that one limitation in qualitative research is the absence of statistical generalization seen in quantitative research. Unlike quantitative research, the primary aim of most qualitative studies is not generalization, but rather to provide a deep, contextual understanding of human experiences through the detailed examination of specific cases [87]. This current study indirectly, rather than directly, recorded and analyzed the human experience, as the researchers interpreted data from other studies in which participants were involved and not from the participants themselves. Qualitative studies propose a form of generalization where the focus is on the phenomenon itself, rather than the entire population [87]. Moreover, grounded theory may sometimes overlook existing theories or literature on a topic, leading to the reinvention of concepts. Finally, theoretical sampling can be a complex procedure, as the selection of the final sample from which the data are extracted may not always be representative [54,55]. For example, in the present study, only the search engines Scopus and Web of Science were used to find relevant articles, and this was undertaken by using a specific BOOLEAN expression. If other search engines, such as PubMed, had been used, or if the BOOLEAN expression had been different, it is possible that additional documents could have been found and used as data for our study.

This grounded theory of the factors that influence the phenomenon of childhood obesity, which combines seven basic concepts, offers a dynamic framework for maximizing the knowledge of the deeper causes influencing the phenomenon of childhood obesity. Through the application of this theory, governments, social structures, hospitals, schools, healthcare systems, and mental health services can, by recognizing the underlying causes that contribute to this phenomenon, implement policies and interventions to address childhood obesity at the core. Moreover, they can enhance social awareness by designing preventive actions and initiatives that will lead to informing and mobilizing populations not only about the seriousness of the issue, but also about ways to control it before it arises.

In summary, this study’s contribution to the literature is that a thorough examination identified a wide variety of factors related to childhood obesity, providing 336 distinct codes that influence its onset, progression, and maintenance. To our knowledge, this is the first study focusing on a such a wide range of factors correlated with childhood obesity by using the grounded theory method. Although grounded theory has been applied in research on childhood obesity, our research has provided a more comprehensive list of possible variables affecting the phenomenon under investigation, relative to prior studies. Existing published research using the grounded theory method has primarily addressed either the perceptions of the parents, children, or other groups concerning this specific issue, or specific demographic groups or geographical places, or specific factors affecting childhood obesity like the role of the parent.

Moreover, a holistic model of the phenomenon was presented by highlighting the way in which the categories interacted with each other. This grounded theory thoroughly explored the causes at both a micro-level and macro-level, ranging from the individual, through to the family, and finally to society at large. Through understanding and applying the principles contained in the seven basic concepts and their subcategories, researchers will be able to assess the risk factors of each family context on a personalized level, allowing them to design and organize personalized interventions accordingly. At the same time, they will be able to address childhood obesity as a social phenomenon requiring intervention and design intervention policies that will affect society as a whole.

## Figures and Tables

**Table 1:** Articles included in the study.

Author-Authors	Year	Title	Types of Factors	Reference Number
Batko, B., Kowal, M., Szwajca, M., and Pilecki, M.	2020	Relationship between biopsychosocial factors, body mass and body composition in preschool children	Biological and psychological factors	[60]
Carnell, S., Kim, Y., and Pryor, K.	2012	Fat brains, greedy genes, and parent power: A biobehavioral risk model of child and adult obesity	Parental and biological factors	[61]
Chatzidaki, E., Chioti, V., Mourtou, L., Papavasileiou, G., Kitani, R.-A., Kalafatis, E., Mitsis, K., Athanasiou, M., Zarkogianni, K., and Nikita, K.	2024	Parenting styles and psychosocial factors of mother–child dyads participating in the ENDORSE digital weight management program for children and adolescents during the COVID-19 pandemic	Parental, social and psychological factors	[62]
Coleman, J. R., Krapohl, E., Eley, T. C., and Breen, G.	2018	Individual and shared effects of social environment and polygenic risk scores on adolescent body mass index	Social and biological factors	[63]
Do, L. M., Larsson, V., Tran, T. K., Nguyen, H. T., Eriksson, B., and Ascher, H.	2016	Vietnamese mother’s conceptions of childhood overweight: Findings from a qualitative study	Parental factors	[64]
Faith, M. S., Berkowitz, R. I., Stallings, V. A., Kerns, J., Storey, M., and Stunkard, A. J.	2006	Eating in the absence of hunger: A genetic marker for childhood obesity in prepubertal boys?	Social factors	[65]
Haire-Joshu, D., and Tabak, R.	2016	Preventing obesity across generations: Evidence for early life intervention	Social and biological factors	[66]
Holmen, T. L., Bratberg, G., Krokstad, S., Langhammer, A., Hveem, K., Midthjell, K., Heggland, J., and Holmen, J.	2014	Cohort profile of the young-HUNT study, Norway: A population-based study of adolescents	Biological and psychological factors	[67]
Iguacel, I., Fernández-Alvira, J. M., Ahrens, W., Bammann, K., Gwozdz, W., Lissner, L., Michels, N., Reisch, L., Russo, P., and Szommer, A.	2018	Prospective associations between social vulnerabilities and children’s weight status. Results from the IDEFICS study	Social factors	[68]
Ji, M. and An, R.	2022a	Parental effects on obesity, smoking, and drinking in children and adolescents: A twin study	Parental factors	[69]
Ji, M. and An, R.	2022b	Parenting styles in relation to childhood obesity, smoking, and drinking: A gene–environment interaction study	Social and biological factors	[70]
Kiefner-Burmeister, A., and Hinman, N.	2020	The role of general parenting style in child diet and obesity risk	Parental factors	[71]
Grube, M., Bergmann, S., Keitel, A., Herfurth-Majstorovic, K., Wendt, V., von Klitzing, K., and Klein, A.M.	2013	Obese parents—obese children? Psychological-psychiatric risk factors of parental behavior and experience for the development of obesity in children aged 0–3: Study protocol	Parental and psychological factors	[72]
Mazzeo, S. E., Mitchell, K. S., Gerke, C. K., and Bulik, C. M.	2006	Parental feeding style and eating attitudes: Influences on children’s eating behavior	Parental and psychological factors	[73]
McDonald, G., Faga, P., Jackson, D., Mannix, J., and Firtko, A.	2005	Mothers’ perceptions of overweight and obesity in their children	Parental factors	[74]
Murrin, C. M., Kelly, G. E., Tremblay, R. E., and Kelleher, C. C.	2012	Body mass index and height over three generations: evidence from the Lifeways cross-generational cohort study	Biological factors	[75]
Oparaocha, E.	2018	Childhood obesity in Nigeria: Causes and suggestions for control	Social factors	[76]
Paul, I. M., Williams, J. S., Anzman-Frasca, S., Beiler, J. S., Makova, K. D., Marini, M. E., Hess, L. B., Rzucidlo, S. E., Verdiglione, N., and Mindell, J. A.	2014	The Intervention Nurses Start Infants Growing on Healthy Trajectories (INSIGHT) study	Biological factors	[77]
Poulain, T., Baber, R., Vogel, M., Pietzner, D., Kirsten, T., Jurkutat, A., Hiemisch, A., Hilbert, A., Kratzsch, J., and Thiery, J.	2017	The LIFE Child study: a population-based perinatal and pediatric cohort in Germany	Biological factors	[78]
Regber, S., Dahlgren, J., and Janson, S.	2018	Neglected children with severe obesity have a right to health: Is foster home an alternative?—A qualitative study	Social and parental factors	[79]
Russell, C. G., and Russell, A.	2018	Biological and psychosocial processes in the development of children’s appetitive traits: Insights from developmental theory and research	Biological, social and psychological factors	[80]
Suder, A., and Chrzanowska, M.	2015	Risk factors for abdominal obesity in children and adolescents from Cracow, Poland (1983–2000)	Biological, social and psychological factors	[81]
Van De Beek, C., Hoek, A., Painter, R. C., Gemke, R. J., Van Poppel, M. N., Geelen, A., Groen, H., Mol, B. W., and Roseboom, T. J.	2018	Women, their offspring and improving lifestyle for better cardiovascular health of both (WOMB project): A protocol of the follow-up of a multicenter randomized controlled trial	Biological, social and parental factors	[82]
Vedanthan, R., Bansilal, S., Soto, A. V., Kovacic, J. C., Latina, J., Jaslow, R., Santana, M., Gorga, E., Kasarskis, A., and Hajjar, R.	2016	Family-based approaches to cardiovascular health promotion	Biological and parental factors	[83]
Zhang, Y., Hurtado, G. A., Flores, R., Alba-Meraz, A., and Reicks, M.	2018	Latino fathers’ perspectives and parenting practices regarding eating, physical activity, and screen time behaviors of early adolescent children: Focus group findings	Parental factors	[84]

## Data Availability

No new data were created or analyzed in this study. Table 1 of this study shows the documents that were used as data for the construction of the theory.

## Appendix A. The Categories, Subcategories, and Codes That Emerged

**CATEGORIES**	**SUBCATEGORIES AND CODES**
CATEGORY 1. SOCIAL FACTORS	RELATED WITH PARENTAL SOCIAL STATUS: Socioeconomic status, Low or medium income, Social class, Occupation of the parents, Economic situation of family, Educational level of the parents (particularly maternal education), Unemployment of the parents, Poverty, Social vulnerabilities, Prolonged maternal full-time employment, Parental unemployment (particularly paternal unemployment, Migrant status, Occupational prestige, Poor quality of life, Parental cognitions
	RELATED WITH SPECIFIC TIME PERIODS: The impact of COVID-19, The impact of the measures for the management of COVID-19, Consumption of cheap and easily available high-calorie food as a lifestyle, Decreased or lack of physical activity as a lifestyle, Lifestyle changes in teenagers, Overconsumption of foods and beverages as a lifestyle, Lack of undertaking physical activity in sport clubs in boys, Change in nutritional habits, Social changes, Generation specific effects, Lifestyle behaviors during pregnancy, Snacking dietary pattern in school children
	RELATED WITH SPECIFIC GEOGRAPHIC LOCATIONS AND CULTURES: Living in rural areas, Poor-quality environments, Early feeding practices supported by family culture, Socioeconomic deprivation during the prenatal period and early childhood, Epidemiologic and demographic transitions, Urbanization, Affluence, Political environment, Failing economic environment, Cultural effects, Social inequality as a result of economic insecurity
	RELATED WITH SPECIFIC IDEOLOGIES: Cultural beliefs that define a larger infant as representing a healthy and active child, Bogus beliefs and taboos, The concept that “chubby children look cute and lovely”, The concept that “overweight is a minor problem”, The concept that “a large infant is an indication of successful mothering”, Low subjective perceptions of social position, Gender inequalities, Gender roles, Women having primary responsibility in food parenting practices and nutrition, Fathers’ and mother’s beliefs and concerns about nutrition and physical activity, Mistakes of the parents on children’s appropriate diet and weight
	RELATED WITH SOCIAL NETWORKS AND OTHER INFLUENCING FACTORS: Lack of support of parents in interventions aimed at the prevention and management of overweight, School environment, Lack of school-based strategies for obesity prevention, Low support from formal and informal sources, Low social support, Minimal social networks, Societal neglect, Lack of guidance of recommended dietary guidelines, Bereavement, Language barrier, Culture shock and lack of acceptance by the new nation in migrant children, Psychosocial stress and feelings of insecurity, Effect of the media, Intergenerational transmission of social disadvantage and health outcomes, Lack of nutritional discipline
CATEGORY 2. GENETIC AND BIOLOGICAL FACTORS	GENETIC FACTORS: Age (greater effects on youngest), Gender (greater genetic effects on boys), Combination of the gender of both parent and child, Child’s birth weight, Familial height and weight, Height, Mother’s age at delivery, The composition of bacteria in the gut, the human microbiome, Genes influencing dopamine and serotonin function, Changes to the precursor stem cell of adipose cells and neurons related to appetite regulation, Epigenetic adaptations and changes, Intergenerational influences, Genetic makeup of individuals, Slow metabolism, Genomics
	BIOLOGICAL FACTORS: Mechanism of metabolic programming, Heredity, Monogenic or endocrine causes, Metabolic pathways, Hormonal signaling, Altered glucose metabolism, Growth trajectory, Epigenetic influences that cause heritable alterations in gene expression, Intergenerational transfer of obesity, Intrauterine environment and biological programming, Developmental origins of disease, Low fat-free mass, Functional connectivity between the ventral striatum and emotion/motor preparation structures, Connectivity between the ventral striatum and amygdala and attention-related regions, Inflammatory markers, Earlier onset of puberty in females
	FACTORS DURING PREGNANCY AND PRENATAL PERIOD: Mother’s diet during pregnancy, Maternal weight gain during pregnancy, Maternal obesity during the first trimester of pregnancy, Excess maternal weight prior to conception, Healthy diet and regular physical activity during pregnancy, Altered metabolism in offspring resulting from variations in the father’s diet, Hormonal signaling during pregnancy, Altered glucose metabolism during pregnancy, Exposure to leptin during the prenatal period, Changes in certain metabolic pathways during pregnancy, Alterations in maternal metabolism, Under- and overnutrition and micronutrient intake during pregnancy, Maternal biology, Gestational diabetes, Greater methylation of specific genes prenatally, Mothers unique influence on offspring body composition, possibly through intrauterine mechanisms, Excessive gestational weight gain (GWG), Rapid infant weight gain, Excess weight at ages 6 months, 1 year, and 2 years, Maternal and parental smoking during the prenatal period, Exercise during pregnancy, The food that a mother consumes and the experiences of taste and smell that function during fetal life, Changes to the placenta, Exercise during pregnancy
	BIOLOGICAL AND OTHER INDICATORS FROM THE PARENTS: Abnormal body mass in at least one of the parents, Obesity in both parents affects boys and girls, Obese parents affect sons, Obese mothers affect daughters, Parents slimness in childhood, Parent’s diet, Taste and nutrition preferences of parents, Parents’ smoking habits affect children and especially girls, Paternal and maternal smoking during pregnancy, Mothers’ nutritional status throughout her life, Food cue responsiveness, Maternal smoking during her life
CATEGORY 3. FAMILY CONDITION-RELATED FACTORS	PSYCHO-EMOTIONAL FACTORS RELATED WITH FAMILY AND PARENTS: Stress-coping styles presented by the mothers, Maternal stress, Lack of the ability of parents to regulate their emotions (sadness, stress, etc.), Child maltreatment, Quality of child care, Instrumental feeding, Emotional feeding, The parents’ experience of stress after the birth of the child and during toddlerhood, Insufficient capacity of mothers to decode nonverbal expressions of emotions, Fathers’ mixed levels of self-efficacy in food and activity parenting practices, Resistance from children as a major barrier to promoting healthy eating and physical activity at home
	FAMILY-MEMBERS RELATIONAL FACTORS: Difficulties in family relationships, Poor family functioning, Home environment factors, Emotional climate during meals, Poor communication, Poor behavior control, High levels of family conflict, Low family hierarchy values, Discord between parents, Violence, Household dysfunction, The role of food in family gatherings, Family cohesion and flexibility, Family food rules or rituals
	COGNITIVE PERCEPTIONS AND BEHAVIORAL FACTORS OF THE PARENTS: Taste and nutrition preferences of parents, Mothers’ nutritional status throughout her life, Parental healthy modeling, Low parental concerns about their child’s thinness, Parental concern about child weight, Parental difficulty in recognizing weight problems, Parental perceptions of the diet, Authoritative feeding style, Authoritarian (restrictive) feeding style, Autonomy-supportive food parenting practices
	PREVAILING FAMILY CONDITIONS: Having only one son in the family, Parental separation or divorce, Living with a substance abuser, Imprisonment of a household member, Witnessing a parent being abused, Living with a mentally ill person, The effect of birth order, Being part of nontraditional families, Number of children in family, Adverse experiences in childhood, Limited time to take care of children
CATEGORY 4. PSYCHOLOGICAL FACTORS	MENTAL HEALTH ISSUES: Depression, Anxiety, Eating disorders, Coping with stress, Infant’s temperament, Autism spectrum disorders, Attention-deficit hyperactivity disorder, Alexithymia, Behavior disorders, Negative emotionality, Negative self-evaluation, Poor self-image, Body dissatisfaction, Conduct problems, Hyperkinetic disorders (hyperactivity, inattention, and impulsivity), Peer relationship problems and prosocial behavior, Coping with stressful situations, Coping with traumatic experiences
	PSYCHOLOGICAL FACTORS CONNECTED WITH FOOD CONSUMPTION: Emotion regulation with food, Disturbing behavior, Neophobia (fear of new foods), Food addiction, Tantrums over food, Delay of gratification, Overeating amongst girls, Binge eating, Emotional feeding from parents, Inability to monitor food intake, Emotional eating, Eating in the absence of hunger, Higher food responsiveness (being attracted to food and eating)
	COPING WITH EMOTIONS ISSUES: Psychological control, Behavioral regulation, Social-emotional competence, Emotion and self-regulation, Inhibitory control, Emotional reactivity, Increased levels of negative affect, Less emotional awareness, Difficulty in coping with negative emotions, Child emotional insecurity, Problems with experiencing, describing, and identifying one’s emotions, Internalizing or externalizing difficulties, Emotional abuse
CATEGORY 5. PARENTING STYLE	GENERAL PARENTING STYLE: Strict parenting style, Authoritative parenting style (Balanced use of open, communicative warmth and assertive discipline), Permissive parenting style (little to no discipline or control over a child), Authoritarian parenting style (Heavy use of control and discipline with little warm communication), Neglectful parenting style, Responsiveness of the parent, Demandingness of the parent (especially of the mother), Uninvolved parenting style, Negative parental practices, Uninvolved parenting style (parents who are low on both warmth and control), Inconsistent parenting, Poor parenting
	RELATED TO EMOTIONAL AND PSYCHOLOGICAL SITUATIONS: Monitoring and controlling child activities and deviant behaviors, Lack of praise, Levels of parental and maternal emotional warmth, Parental psychological control, Family communication, Negative paternal and maternal communication, Parental neglect, Insecure attachment relationship, Lack of acceptance from the parents, Poor mother–child relationship followed by an insecure mother–child attachment, Parental interpersonal dysphoria, Maternal intrusiveness, Levels of parental support and encouragement, Overprotection, Coercive control, Differential parental treatment to the kids of a family, Soothing strategies for infant/toddler distress and fussiness, Parental responsiveness to their child’s needs, Absent parents, Maternal depression, self-esteem, financial strain, and maternal distress
CATEGORY 6. FEEDING AND HEALTH RELATED PRACTICES	PRACTICES AROUND FOOD CONSUMPTION: Eating habits such as not drinking enough water, or not chewing food adequately, Not offering assistance during mealtimes, Early introduction of complementary solid foods, Exposure to a certain food type after a period of restriction to it, Pressing the children to eat, Not promoting self-regulation of the children, Parental strict limitations in food, Food fussiness, Absence of frequent family meals, Formula-fed infants, Age-inappropriate feeding, Greater role for fat and added sugars in foods, Reduced intakes of complex carbohydrates and dietary fiber, Reduced fruit and vegetable intake, Eating rate, Disinhibited eating, Use of food as a reward, Large portions, Response to children’s hunger and fullness cues, Breastfeeding period
	HEALTH RELATED PRACTICES: Not enhancing physical activity, Not controlling screen time, Absence of establishment of rules for sleep schedules, Absence of age-appropriate sleep patterns and duration, Enhancing sedentary behavior, Use of car seats and strollers, Exposure to television and media, Sleep deprivation, Having a television in children’s bedrooms, Quality of sleep, Medication, Having the television on during dinner, Leisure time activities, Drug, alcohol, cigarette consumption, Not doing things together with children, Spending time with children in physical activities
	PRACTICES AROUND FOOD PREPARATION AND AVAILABILITY: Availability of healthy food at home, Not educating children about nutrition, No involvement of the children in preparing meals, Not offering different choices for food consumption, Not discussing food choices with children, Absence of flexible, individualized dietary plan, Absence of clear and consistent rules related to food, Not respecting infant’s or toddler’s flavor or food preferences, Not respecting appetitive characteristics and traits, Allowing children unrestricted access to inappropriate foods or displaying no supportive guidance, Asserting strict control over all feeding behaviors, Not enhancing the children to eat both new and familiar foods, Intake of unhealthy snack foods as an easy choice
CATEGORY 7. CONSEQUENCES OF OBESITY	SOCIAL: Weigh related stigma, Body image concerns, Being avoided, ignored, or the subject of negative rumors, Problems of integration with peers, Bullying, Joint problems, Dissatisfaction with one’s own body, High school drop-out, Reduced work integration, Poor quality of life
	PSYCHOLOGICAL: Emotional difficulty, Mental disorders, Higher rates of sadness, loneliness, and nervousness, Decreased self-esteem, Psychological problems, Poor self-image, Depression, Anxiety, Psychiatric health problems, Suicidality, Poorer well-being
	BIOLOGICAL: Increases mortality, Sixth risk factor for death, Cardiovascular disorders, Metabolic disorders, Adult obesity, Diabetes and insulin resistance, Renal and liver disorders, Musculoskeletal disorders, Respiratory disorders, Neurological disorders, Chronic diseases, Menstrual disorders, Fertility challenges, Cancers of the esophagus, pancreas, colon and rectum, breast (post-menopausal), endometrium, and kidney, Lower physical functioning performance, High blood pressure, Asthma
